# Cold weather increases respiratory symptoms and functional disability especially among patients with asthma and allergic rhinitis

**DOI:** 10.1038/s41598-018-28466-y

**Published:** 2018-07-04

**Authors:** Henna Hyrkäs-Palmu, Tiina M. Ikäheimo, Tiina Laatikainen, Pekka Jousilahti, Maritta S. Jaakkola, Jouni J. K. Jaakkola

**Affiliations:** 1 Center for Environmental and Respiratory Health Research, University of Oulu, P.O. Box 5000, FI-90014 Oulu, Finland; 2 Medical Research Center, University of Oulu and Oulu University Hospital, Oulu, Finland; 3 National Institute for Health and Welfare, Public Health Solutions, FI-00271 Helsinki, Finland; 4 Institute of Public Health and Clinical Nutrition, University of Eastern Finland, FI-70211 Kuopio, Finland; 5 Joint municipal authority for North Karelia social and health services (Siun sote), FI-80210 Joensuu, Finland

## Abstract

Cold weather affects the respiratory epithelium and induces bronchial hyperresponsiveness. We hypothesized that individuals with allergic rhinitis or/and asthma experience cold weather-related functional disability (FD) and exacerbation of health problems (EH) more commonly than individuals without these. This was a population-based study of 7330 adults aged 25–74 years. The determinants of interest, including doctor-diagnosed asthma and allergic rhinitis, and the outcomes, including cold weather-related FD and EH, were measured using a self-administered questionnaire. The prevalences of cold-related FD and EH were 20.3% and 10.3%, respectively. In Poisson regression, the risk of FD increased in relation to both allergic rhinitis (adjusted prevalence ratio (PR) 1.19, 95% CI 1.04–1.37 among men; 1.26, 95% CI 1.08–1.46 among women), asthma (1.29, 0.93–1.80; 1.36, 0.92–2.02, respectively) and their combination (1.16, 0.90–1.50; 1.40, 1.12–1.76, respectively). Also the risk of cold weather-related EH was related to both allergic rhinitis (1.53, 1.15,−2.04 among men; 1.78, 1.43–2.21 among women), asthma (4.28, 2.88–6.36; 3.77, 2.67–5.34, respectively) and their combination (4.02, 2.89–5.59; 4.60, 3.69–5.73, respectively). We provide new evidence that subjects with allergic rhinitis or/and asthma are more susceptible to cold weather-related FD and EH than those without pre-existing respiratory diseases.

## Introduction

Asthma and allergic rhinitis are common chronic diseases worldwide^1^. The prevalence of doctor-diagnosed asthma has been estimated as 4.3% globally and it has been increasing, especially in the developed countries^2^. The prevalence of allergic rhinitis has been estimated to be even higher in European populations, i.e. 30.9% in 2008 in the Swedish population^3^ and 37.4% in 2011 in the Italian population^4^. In Finland, both asthma and allergic rhinitis have been increasing during the past decades. The prevalence of asthma among adult population was 8.5–11.2% and the prevalence of allergic rhinitis/hay fever 23.0–38.5% in 2012^5^. Asthma and allergic rhinitis have been found to be strongly linked to each other^6^. Previous epidemiological studies have shown that respiratory symptoms such as wheezing, cough and phlegm production are common among adults in all age groups^3,4^.

We hypothesized that cold weather causes functional disabilities among individuals with existing respiratory disease. This is because low temperature and accompanying low air humidity are likely to affect the respiratory epithelium and induce hyperresponsiveness and narrowing of the respiratory airways^7,8^. Cold weather may further aggravate symptom reporting and especially among those who have some underlying respiratory diseases^9^. There is also previous evidence that those whose asthma is in poor control are more prone to cold weather-related respiratory symptoms than those whose asthma is in good control^10^.

Cooling and drying of respiratory epithelium may induce chronic inflammation, which is likely to increase respiratory symptoms. Exercising in cold weather is likely to further strengthen the experience of respiratory symptoms and functional disability^11^.

Cold weather-related functional disabilities among asthmatics have not been studied at the population level. There are a few studies that have shown decreasing lung function related to exercising or working in cold outdoor weather^12,13^. Decline of lung function and high prevalence of respiratory symptoms in cold indoor environment has also been reported^14,15^. A Finnish study of 14 patients demonstrated that those who had chronic obstructive pulmonary disease (COPD) experienced a decrease in exercise performance in cold air^16^.

The objective of the present study was to investigate potential relations between cold temperature and reported functional disability and exacerbation of health problems among subjects with pre-existing respiratory diseases, i.e. asthma and allergic rhinitis. We hypothesized that subjects with asthma and/or allergic rhinitis experience more cold weather-related functional disabilities than subjects without such underlying respiratory diseases, and that cold weather exacerbates their health problems.

The study population was from two population-based studies conducted in 2007 (FINRISK 2007) and 2012 (FINRISK 2012). A stratified random sample of 25–74 year old subjects was drawn from the Finnish population register.

## Results

### Characteristics of the study population

The study comprised 7330 participants from FINRISK 2007 and FINRISK 2012. Table 1 presents the characteristics of the study population according to gender. More than half of the subjects (57.5%) were 50 years old or older, and almost one fourth of the subjects were obese or severely obese (23.3%). Almost half of the subjects (48.5%) were indoor workers (office workers, students etc.).

**Table 1:** Characteristics of the study population, FINRISK 2007 & 2012.

Characteristic	Men n (%) 3369 (46.0)	Women n (%) 3961 (54.0)	Total n (%) 7330
**Age (years)**			
<30	226 (6.7)	347 (8.8)	573 (7.8)
30–39	506 (15.0)	645 (16.3)	1151 (15.7)
40–49	625 (18.6)	769 (19.4)	1394 (19.0)
50–59	744 (22.1)	905 (22.9)	1649 (22.5)
>60	1268 (37.6)	1295 (32.7)	2563 (35.0)
**Body mass index (kg/** **m** ^**2**^ **)**			
<20	40 (1.2)	200 (5.1)	240 (3.3)
20–25	940 (27.9)	1524 (38.5)	2464 (33.6)
25–30	1629 (48.4)	1287 (32.5)	2916 (39.8)
30–35	570 (16.9)	610 (15.4)	1180 (16.1)
>35	190 (5.6)	339 (8.6)	529 (7.2)
Missing	0	1	1
**Marital status**			
Single	473 (14.1)	488 (12.3)	961 (13.1)
Marriage/Cohabitation	2559 (76.1)	2755 (69.6)	5314 (72.6)
Divorced, separated or widow	332 (9.9)	714 (18.0)	1046 (14.3)
Missing	5	4	9
**Education**			
Low	1060 (31.6)	1081 (27.4)	2141 (29.3)
Medium	1591 (47.4)	1826 (46.3)	3417 (46.8)
High	708 (21.1)	1034 (26.2)	1742 (23.9)
Missing	10	20	30
**Work**			
Agriculture, etc.	824 (24.7)	208 (5.3)	1032 (14.2)
Office, studies	1274 (38.2)	2247 (57.2)	3521 (48.5)
Housewife, retired, unemployed	1239 (37.1)	1473 (37.5)	2712 (37.3)
Missing	32	33	65
**Smoking**			
Current smoker	740 (22.1)	642 (16.3)	1382 (19.0)
Ex-smoker	1106 (33.1)	771 (19.5)	1877 (25.8)
Never smoker	1498 (44.8)	2532 (64.2)	4030 (55.3)
Missing	26	15	41
**Second hand smoke exposure**			
Yes	322 (9.9)	191 (5.0)	513 (7.3)
No	2926 (90.1)	3634 (95.0)	6560 (92.8)
Missing	121	136	257

Education categories: Low = comprehensive or upper secondary school degree, Medium = vocational or upper secondary and vocational school, High = higher vocational or academic.

**Figure 1:**
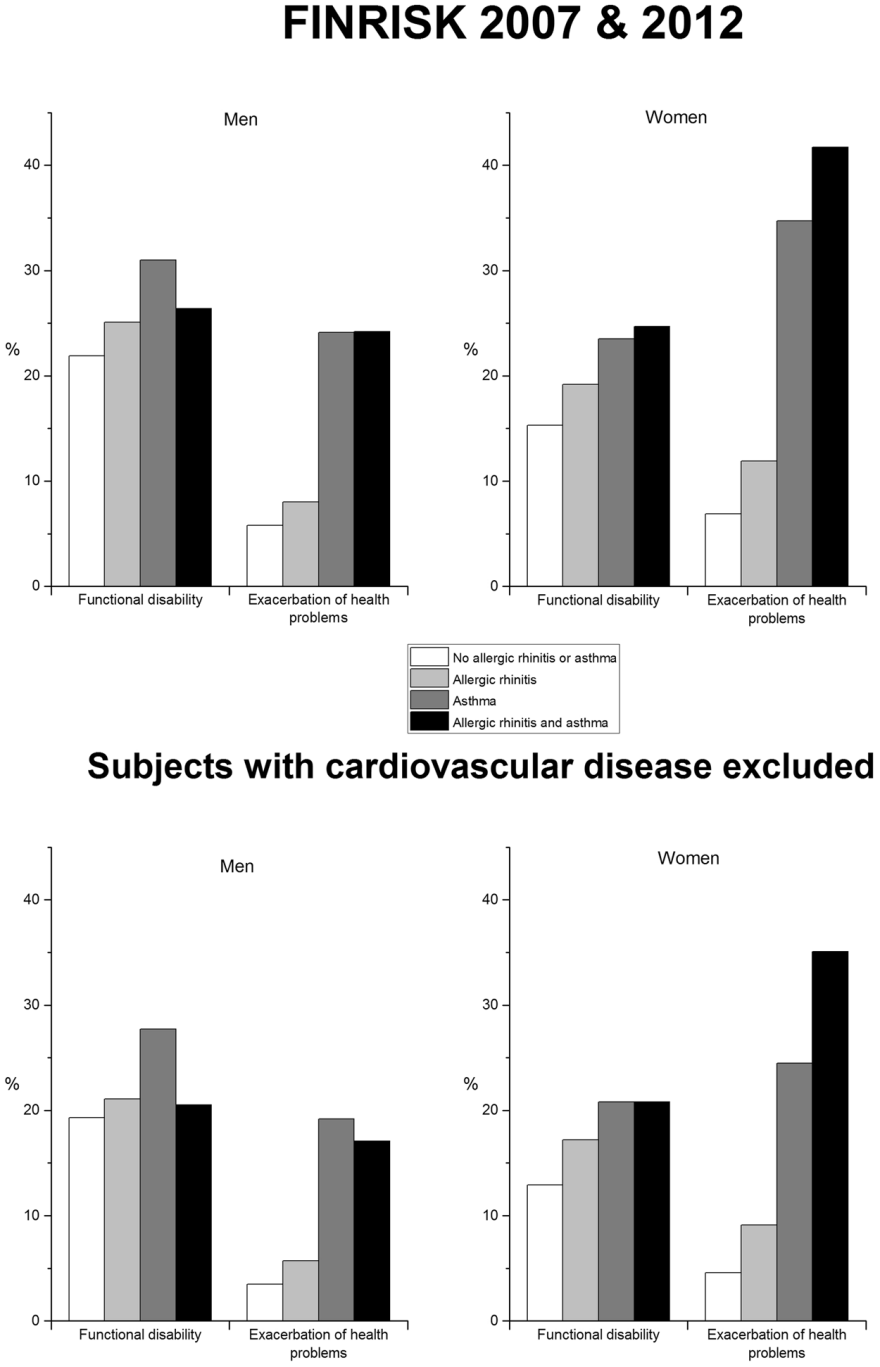
Cold weather-related FD and EH according to having allergic rhinitis, asthma, or both of them.

Table 2 presents the distributions among the whole study population and among those without any cardiovascular disease. The prevalences of the determinants of interest were somewhat similar between the whole population and the sub-population (Fig. 1). In the whole study population, there were slightly more asthmatics and slightly less those with only allergic rhinitis compared with the sub-population.

**Table 2:** The distribution of the determinants of interest in the total study population and the sub-population.

	FINRISK 2007 & 2012			No cardiovascular diseases		
	Men n (%)	Women n (%)	Total n (%)	Men n (%)	Women n (%)	Total n (%)
No asthma or allergic rhinitis	2143 (64.0)	2200 (55.6)	4343 (59.5)	1138 (64.4)	1297 (57.0)	2435 (60.2)
Allergic rhinitis without asthma	937 (28.0)	1311 (33.2)	2248 (30.8)	494 (28.0)	768 (33.8)	1262 (31.2)
Asthma without allergic rhinitis	87 (2.6)	103 (2.6)	190 (2.6)	47 (2.7)	54 (2.4)	101 (2.5)
Asthma with allergic rhinitis	184 (5.5)	340 (8.6)	524 (7.2)	88 (5.0)	156 (6.9)	244 (6.0)
Missing	18	7	25	11	2	13

### Cold weather-related functional disability in relation to allergic rhinitis and/or asthma

Table 3 presents the prevalence of cold weather-related FD according to the presence or absence of allergic disease and asthma, separately among men and women. It also shows crude and adjusted prevalence ratios of FD among subjects with asthma and/or allergic diseases in comparison with the reference group of subjects without these allergic diseases.

**Table 3:** Prevalences (%) and prevalence ratios (PR) and their 95% confidence intervals (95% CI) of cold weather-related FD according to having asthma with or without allergic rhinitis or allergic rhinitis alone, FINRISK 2007 & 2012.

Determinant category	FINRISK 2007 & 2012					
	Men			Women		
	No of subjects and prevalence n (%)	Crude PR (95% CI)	Adjusted PR (95% CI)	No of subjects and prevalence n (%)	Crude PR (95% CI)	Adjusted PR (95% CI)
No asthma or allergic rhinitis (reference)	462 (21.9)	1.00	1.00	330 (15.3)	1.00	1.00
Allergic rhinitis without asthma	233 (25.1)	**1.15 (1.00–1.32)**	**1.19 (1.04–1.37)**	249 (19.2)	**1.26 (1.08–1.46)**	**1.26 (1.08–1.46)**
Asthma without allergic rhinitis	27 (31.0)	**1.42 (1.03–1.96)**	1.29 (0.93–1.80)	23 (23.5)	**1.54 (1.06–2.23)**	1.36 (0.92–2.02)
Asthma with allergic rhinitis	48 (26.4)	1.21 (0.94–1.55)	1.16 (0.90–1.50)	83 (24.7)	**1.62 (1.31–2.00)**	**1.40 (1.12–1.76)**
Missing	62 (1.8)			63 (1.6)		

Adjusted PR for age, BMI, marital status, education, job category, smoking, exposure to secondhand smoke (SHS) and other diseases.

Among the total study population, 20.3% of the participants reported FD related to cold weather. Among the total study population, women reported it slightly less commonly (17.6%) compared to men (23.4%). The prevalence of FD was lowest in the reference group both in men (21.9%) and in women (15.3%). Subjects with allergic rhinitis alone experienced cold weather-related FD more commonly (25.1% and 19.2%) with an adjusted PR of 1.19 (95% CI 1.04–1.37) among men and 1.26 (95% CI 1.08–1.46) among women. The presence of asthma increased cold weather-related FD slightly more, giving an adjusted PR of 1.29 (95% CI 0.93–1.80) for men and 1.36 (95% CI 0.92–2.02) for women. The presence of allergic rhinitis in addition to asthma did not increase the effect estimates, resulting in similar adjusted PRs as those who had asthma only, the estimates being 1.16 (0.90–1.50) and 1.40 (1.12–1.76), respectively.

Supplementary Tables S1 and S2 shows also the results from the analyses focusing on subjects without cardiovascular disease. The effect estimates were similar or slightly greater in this sub-population.

### Cold weather-related impairment of health and exacerbation of disease symptoms in relation to allergic rhinitis and/or asthma

Table 4 presents the prevalences of cold weather-related EH according to the presence or absence of allergic disease and asthma. The overall prevalence was 10.3%, 7.9% among men and 12.3% among women. Allergic rhinitis alone increased the experience of cold weather-related EH significantly with an adjusted PR of 1.53 (1.15–2.04) in men and 1.78 (1.43–2.21) in women. Moreover, the presence of asthma without allergic rhinitis was a substantially stronger determinant, with an adjusted PR of 4.28 (2.88–6.36) in men and 3.77 (2.67–5.34) in women. As shown in Table 4, allergic rhinitis in addition to asthma did not influence much the effect estimate among men (adjusted PR of 4.02 (2.89–5.59)), but among women the adjusted PR was 4.60 (3.69–5.73). The results of the sensitivity analyses indicated similar or greater effect estimates in the sub-population of subjects without any cardiovascular diseases.

**Table 4:** Prevalences (%), prevalence ratios (PR) and their 95% confidence intervals (CI) of cold weather-related EH according to having asthma with or without allergic rhinitis, and allergic rhinitis alone, FINRISK 2007 & 2012.

Determinant category	FINRISK 2007 & 2012					
	Men			Women		
	No of subjects and prevalence n (%)	Crude PR (95% CI)	Adjusted PR (95% CI)	No of subjects and prevalence n (%)	Crude PR (95% CI)	Adjusted PR (95% CI)
No asthma or allergic rhinitis (reference)	123 (5.8)	1.00	1.00	149 (6.9)	1.00	1.00
Allergic rhinitis without asthma	74 (8.0)	**1.37 (1.04–1.81)**	**1.53 (1.15–2.04)**	155 (11.9)	**1.73 (1.40–2.14)**	**1.78 (1.43–2.21)**
Asthma without allergic rhinitis	21 (24.1)	**4.14 (2.75–6.24)**	**4.28 (2.88–6.36)**	34 (34.7)	**5.04 (3.69–6.89)**	**3.77 (2.67–5.34)**
Asthma with allergic rhinitis	44 (24.2)	**4.15 (3.05–5.65)**	**4.02 (2.89–5.59)**	140 (41.7)	**6.05 (4.96–7.39)**	**4.60 (3.69–5.73)**
Missing	62 (1.8)			63 (1.6)		

Adjusted PR for age, BMI, marital status, education, job category, smoking, exposure to secondhand smoke (SHS) and other diseases.

## Discussion

Our results show that both perceived functional disability and exacerbation of health problems related to cold weather are common in the Finnish adult population. A considerable amount of both men (18%) and women (23%) reported functional disability, and approximately 6% of men and 7% of women expressed exacerbation of health problems related to cold weather. Moreover, both allergic rhinitis alone and in combination with asthma were associated with higher reporting of cold weather-related functional disability. The impact of cold weather was substantially stronger among subjects with allergic rhinitis and even stronger among subjects with asthma alone. Allergic rhinitis in combination to asthma did not increase the impact any more compared with asthma. There were no major differences in the effects of cold weather between men and women.

In this study, one fifth of the study population reported cold weather-related functional disability. This can be a consequence of the physiological changes linked to the cooling effect in the airways^7,17^, cardiovascular system^18^, and/or muscular system^19^. These can cause reduction in cognitive^20^ and/or physical performance^21^, and through that increase the perceived functional disability. Occupational studies have indicated that daily exposure of cold air alters lung function^14^ and can cause musculoskeletal pain^19^. The interpretation of the term “functional disability” is likely to vary broadly between individuals. Functional disability is likely to be related to the effects of cold weather on both physical and cognitive performance. To our knowledge, this is the first study that investigates perceived functional disability on population level, and investigates its prevalence among those who have allergic rhinitis or/and asthma.

Our study shows that especially those with asthma alone or in combination with allergic rhinitis reported exacerbation of health problems in cold weather. This follows the same trend as our previous study among young adults, which indicated that those who had allergic rhinitis and asthma alone or in combination experienced more cold weather-related respiratory symptoms than healthy subjects^22^. A previous population-based study has also shown that among adults, those who have some respiratory disease experience more respiratory symptoms in cold than healthy individuals^9^. Cold air affects airways by causing cooling and drying. Rhinorrhea, congestion and sneezing are common short-term responses to cold air nasal breathing. Moreover, it has been suggested that these responses are greater among those with rhinitis alone and even greater among those with asthma and rhinitis than in healthy subjects^23^. Some studies investigating nasal responses have shown that there is activation of nasal sensory nerve and mast cells^7^. Exposure to cold air has been shown to decrease respiratory function and increase inflammatory markers in sputum^17^. Some previous studies have also suggested that repeated exposure to cold air may have permanent effects on airways through damaging the airway epithelium and altering the airway wall structure and function^11,23^.

Previous studies have suggested that asthma and allergic rhinitis frequently co-exist together in the same individuals^24^, which is consistent with our results. Some studies have suggested that there may be bidirectional link between upper and lower airways and the united airways –theory has been established^25^. This theory suggests that there are some pathophysiological mechanisms that are the same in both diseases and that treatment for allergic rhinitis is beneficial also for the asthma control reducing asthma-related exacerbations and hospitalizations^26^.

Quality of life has been studied among asthmatics. Those who had coexisting allergic rhinitis had worse quality of life than those with asthma alone^27^. Very frequent disease symptoms can also cause functional disability and a Canadian study reported that almost half of the people with allergic rhinitis had year-round symptoms^28^. Allergic rhinitis has not been considered as a serious disease, but previous studies have suggested that it has several effects on everyday life, for example it may cause exhaustion during the day, and it may also have socioeconomic consequences because of absence from school or work^26^.

A previous study showed that there are some overlapping of symptom reporting among people with different kinds of diseases^29^. We included other chronic diseases as a covariate among the whole population in the adjusted analyses. In addition, stratified analyses were conducted for the sub-population by excluding those with cardiovascular diseases, to see if that influences the results. The stratified analyses did not affect the main effects.

The National FINRISK Study is population-based and has an extensive geographical coverage in Finland. The diseases of interest were self-reported doctor-diagnosed asthma and allergic rhinitis. Information on doctor-diagnosed asthma is most likely valid due to substantial reimbursement that is given nationally for asthma medication for patients with a verified diagnosis based on agreed criteria. The assessment of allergic rhinitis was more likely to include measurement error with potential under-diagnosis. This could partly explain the weaker effects among those with allergic rhinitis only. The outcomes of interest were perceived cold weather-related functional disability and exacerbation of health problems. The responses to questions about cold-related phenomena are clearly based on subjective perceptions of cold, although individuals are likely to verify their perceptions against objective temperature. Environmental low temperature is known to be associated with cooling of the body and physiological responses, which elicit discomfort, decreased physical and cognitive performance and worsen the course of chronic diseases (manifested as increased reporting of various symptoms). The study questions were developed to assess these effects. The comprehension of the questions about perceptions of cold reflects both sensory, psychological, environmental, individual and motivational factors. These outcomes are based on self-assessment and may be related to some random error rather than linked to the determinants of interest. Such random error may introduce some underestimation of the studied relations.

Potential determinants of cold weather-related functional disability and health problems could serve as potential confounders. We applied Poisson regression analysis to adjust for several potential confounders, including age, BMI, marital status, education, job category, smoking, exposure to secondhand smoke (SHS) and other diseases. We judged that the presence of a cardiovascular disease could influence the studied outcomes and conducted a sensitivity analysis to explore their impact on the studied effect estimates. We conducted stratified analyses in a sub-population where all subjects with one or more cardiovascular diseases were excluded. The effect estimates for the cold weather-related symptoms were either similar or slightly stronger compared with those estimated in the total population.

Cold weather-related functional disability can cause substantial burden to patients who have these diseases separately or in combination. It is also likely to cause economic burden to society through sick leaves and absence from school or work. This study gives new knowledge for the healthcare personnel who should give advice to those with respiratory diseases to protect themselves from cold weather and to make some changes into their medication. For example, The Finnish Current Care Guidelines for Asthma^30^ include instructions to avoid environmental exposures, which may worsen asthma control and there are instructions for modification of asthma medication. Interestingly, air pollution is indicated as a common irritant but there is no mention about weather conditions in general or cold weather in particular. This suggests that the impact of cold is not recognized among clinicians. According to the present results, the impact of cold weather is rather common among subjects with asthma and allergic rhinitis. It is likely that appropriate modification of medical treatment of asthma and allergies, such as dosage of medication or use of protective mask, would reduce exacerbation of the asthma-related and allergic symptoms and would support ability to cope with the cold weather.

In conclusion, our results show that subjects with allergic rhinitis or/and asthma are more susceptible to cold weather-induced functional disability and exacerbation of symptoms of the pre-existing diseases than subjects without these diseases. Having both allergic rhinitis and asthma did not have an added effect to perceived functional disability and exacerbation of health symptoms compared to having allergic rhinitis or asthma alone.

## Methods

### Study design and study population

The data analyzed in this study consisted of the National FINRISK 2007 and FINRISK 2012 studies conducted by the National Institute for Health and Welfare in Finland. The FINRISK 2007 and 2012 surveys were carried out for a stratified random sample of 25–74 year old population drawn from the Finnish population register^31^. Finland is located between 60° and 70°N latitude and 20° and 31°E longitude. Study areas in the FINRISK 2007 were North Karelia and Northern Savo, Turku and Loimaa regions in southwestern Finland, cities of Helsinki and Vantaa, provinces of Northern Ostrobothnia and Kainuu, and province of Lapland. FINRISK 2012 had the same study areas except the province of Lapland. The monthly mean temperature in 2012 varied in the city of Helsinki from −6.8 °C to 17.7 °C and from −15.8 °C to 13.9 °C in the municipality of Sodankylä located in the province of Lapland. A random sample of the main study population was invited to participate in the temperature-related sub-studies conducted during each survey. Those who agreed to participate also answered The Oulu Cold and Health Questionnaire, which was designed by the specialist study team^32^. This questionnaire data was linked to the FINRISK main study on an individual basis. The Coordinating Ethics Committee of the Helsinki and Uusimaa Hospital District in southern Finland approved studies. All participants of this study signed an informed consent and all methods were performed in accordance with relevant guidelines and regulations.

### Determinants of interest

The respiratory diseases of interest were doctor-diagnosed asthma and allergic rhinitis. Potential occurrence of these diseases were asked in the main FINRISK questionnaires. The potential susceptibility groups investigated were formed of those with only allergic rhinitis, those with only asthma, and those with both allergic rhinitis and asthma. The reference category consisted of those with neither asthma nor allergic rhinitis.

### Outcomes

The main outcomes of interest were cold weather-related functional disability (FD) and impaired health and exacerbation of respiratory symptoms (EH). To address these, the following question was asked: “In which situations do you feel cold as a disability in wintertime? (home inside, home outside, commuting, work outside, work inside, during hobbies or leisure time, or never)”. If cold was felt as a disability in some situation, the following details were inquired: “How serious are these disabilities? (a) feeling uncomfortable, (b) experiencing functional disability, and/or (c) feeling that cold air impairs health or exacerbates symptoms of the disease”.

### Covariates

The following covariates were adjusted for as potential confounders of the studied relations by applying the multivariate analyses: age, body mass index (BMI), marital status, education, type of work, smoking, second hand smoke exposure, and occurrence of other chronic diseases, such as cardiovascular diseases, hypertension, diabetes, musculoskeletal disorders, depression, and other chronic diseases (apart from asthma and allergic rhinitis that were studied as potentially conveying susceptibility to cold). Age and BMI were fitted into 5 categories. The age categories were <30, 30–39, 40–49, 50–59, and >59, where the latter category >59 formed the reference category. BMI was calculated from the weight and height that were measured in a personal check-up at the survey site. BMI categories were <20, 20–25, 25–30, 30–35, and >35, while BMI = 20–25 formed the reference category. Smoking, education, marital status, and type of work were all categorized into 3 groups. For smoking, never smokers formed the reference category, the other categories being ex-smokers and current smokers. For education, higher vocational or academic degree formed the reference category, the other categories being comprehensive or upper secondary school degree, and vocational or upper secondary and vocational school degrees. For marital status, cohabitation/married formed the reference category, while single or separated, and divorced or a widow were the other two categories. Work was categorized to capture potential occupational exposures. Office workers and students formed the reference category, while the other two categories were formed of agriculture, factory work and other jobs with potential asthmogenic exposures, and housewife, retired or unemployed formed the third category.

### Statistical methods

Separate analyses were carried out for men and women. We estimated the relations between the presence of asthma and/or allergic rhinitis and occurrence of the cold weather-related FD and EH, by applying prevalence ratios (PR) and their 95% confidence intervals (95% CI). To address potential susceptibility to cold conveyed by having asthma and/or allergic rhinitis, we estimated the prevalence ratio of the outcomes among these disease groups separately. We also conducted sensitivity analyses by excluding 3275 subjects with a cardiovascular disease. Analyses were conducted applying the SAS statistical program (SAS 9.4, SAS Institute, Inc., Cary, North Carolina). The multivariate analyses were carried out using the GENMOD-procedure, and they were based on Poisson regression using logarithmic link function.

The datasets generated during and/or analyzed during the current study are available from the corresponding author on reasonable request.

## Electronic supplementary material

Supplementary information 1
