# Supplementary material for: Cold weather increases respiratory symptoms and functional disability especially among patients with asthma and allergic rhinitis
Source: Sci Rep. 2018 Jul 4;8:10131. doi: 10.1038/s41598-018-28466-y (PMC6031646; doi:10.1038/s41598-018-28466-y)
Supplement: Supplementary file 1 — Supplementary information 1 [file 41598_2018_28466_MOESM1_ESM.docx]

**Cold weather increases respiratory symptoms and functional disability especially among patients with asthma and allergic rhinitis**

**Authors:**

Henna Hyrkäs-Palmu^a,b^, Tiina M. Ikäheimo^a,b^, Tiina Laatikainen^c,d,e^, Pekka Jousilahti^c^, Maritta S. Jaakkola^a,b^, Jouni J.K. Jaakkola^a,b, *^

^a^ Center for Environmental and Respiratory Health Research, University of Oulu, P.O.Box 5000, FI-90014 Oulu, Finland

^b^ Medical Research Center, University of Oulu and Oulu University Hospital, Finland

^c^ National Institute for Health and Welfare, Public Health Solutions, FI-00271 Helsinki, Finland

^d^ Institute of Public Health and Clinical Nutrition, University of Eastern Finland, FI-70211 Kuopio, Finland

^e^ Joint municipal authority for North Karelia social and health services (Siun sote), FI-80210 Joensuu, Finland

**Correspondence:**

Professor Jouni J. K. Jaakkola

Center for Environmental and Respiratory Health Research,

University of Oulu, P.O.Box 5000, FI-90014 Oulu, Finland

e-mail: jouni.jaakkola@oulu.fi

**Supplementary Information**

Supplementary Table S1

**Table S1 Prevalences (%) and prevalence ratios (PR) and their 95% confidence intervals (95% CI) of cold weather-related FD according to having asthma with or without allergic rhinitis or allergic rhinitis alone, subjects with cardiovascular disease excluded.** Adjusted PRs for age, BMI, marital status, education, job category, smoking, exposure to secondhand smoke (SHS) and other diseases.

|  | **FINRISK 2007 & 2012**, **Subjects with cardiovascular disease excluded** | | | | | |
| --- | --- | --- | --- | --- | --- | --- |
|  | Men | | | Women | | |
| Determinant category | No of subjects and prevalence (%) | Crude PR  (95% CI) | Adjusted^a^ PR (95% CI) | No of subjects and prevalence (%) | Crude PR  (95% CI) | Adjusted^a^ PR (95% CI) |
| No asthma or allergic rhinitis (reference) | 218 (19.26) | 1.00 | 1.00 | 166 (12.93) | 1.00 | 1.00 |
| Allergic rhinitis without asthma | 104 (21.14) | 1.10 (0.89, 1.35) | 1.12 (0.91, 1.38) | 132 (17.23) | **1.33 (1.08, 1.65)** | **1.32 (1.06, 1.64)** |
| Asthma without allergic rhinitis | 13 (27.66) | 1.44 (0.89, 2.31) | 1.48 (0.93, 2.37) | 11 (20.75) | 1.61 (0.93, 2.76) | 1.68 (0.99, 2.85) |
| Asthma with allergic rhinitis | 18 (20.45) | 1.06 (0.69, 1.63) | 1.13 (0.74, 1.74) | 32 (20.78) | **1.61 (1.14, 2.26)** | 1.44 (0.98, 2.10) |
| Missing | 19 (1.1) |  |  | 20 (0.9) |  |  |

Supplementary Table S2

**Table S2 Prevalences (%), prevalence ratios (PR) and their 95% confidence intervals (CI) of cold weather-related EH according to having asthma with or without allergic rhinitis, and allergic rhinitis alone, subjects with cardiovascular disease excluded.** Adjusted PRs for age, BMI, marital status, education, job category, smoking, exposure to secondhand smoke (SHS) and other diseases.

|  | **FINRISK 2007 & 2012, Subjects with cardiovascular disease excluded** | | | | | |
| --- | --- | --- | --- | --- | --- | --- |
|  | Men | | | Women | | |
| Determinant category | No of subjects and prevalence (%) | Crude PR  (95% CI) | Adjusted^a^ PR (95% CI) | No of subjects and prevalence (%) | Crude PR  (95% CI) | Adjusted^a^ PR (95% CI) |
| No asthma or allergic rhinitis (reference) | 40 (3.53) | 1.00 | 1.00 | 59 (4.60) | 1.00 | 1.00 |
| Allergic rhinitis without asthma | 28 (5.69) | **1.61 (1.01, 2.58)** | 1.53 (0.95, 2.49) | 70 (9.14) | **1.99 (1.42, 2.78)** | **2.07 (1.47, 2.92)** |
| Asthma without allergic rhinitis | 9 (19.15) | **5.42 (2.80, 10.50)** | **5.46 (2.77, 10.78)** | 13 (24.53) | **5.34 (3.13, 9.10)** | **5.61 (3.20, 9.82)** |
| Asthma with allergic rhinitis | 15 (17.05) | **4.82 (2.77, 8.39)** | **4.15 (2.30, 7.49)** | 54 (35.06) | **7.63 (5.50, 10.58)** | **6.40 (4.41, 9.29)** |
| Missing | 19 (1.1) |  |  | 20 (0.9) |  |  |
